# Metaproteomics analysis of the functional insights into microbial communities of combined hydrogen and methane production by anaerobic fermentation from reed straw

**DOI:** 10.1371/journal.pone.0183158

**Published:** 2017-08-17

**Authors:** Xuan Jia, Bei-Dou Xi, Ming-Xiao Li, Yang Yang, Yong Wang

**Affiliations:** 1 Key Laboratory of Cleaner Production and Integrated Resource Utilization of China National Light Industry, Beijing Technology and Business University, Beijing, China; 2 State Key Laboratory of Environmental Criteria and Risk Assessment, Chinese Research Academy of Environmental Sciences, Beijing, China; Hubei University, CHINA

## Abstract

A metaproteomic approach was used to analyse the proteins expressed and provide functional evidence of key metabolic pathways in the combined production of hydrogen and methane by anaerobic fermentation (CHMP-AF) for reed straw utilisation. The functions and structures of bacteria and archaea populations show significant succession in the CHMP-AF process. There are many kinds of bacterial functional proteins, mainly belonging to phyla *Firmicutes*, *Proteobacteria*, *Actinobacteria* and *Bacteroidetes*, that are involved in carbohydrate metabolism, energy metabolism, lipid metabolism, and amino acid metabolism. Ferredoxin-NADP reductase, present in bacteria in genus *Azotobacter*, is an important enzyme for NADH/NAD^+^ equilibrium regulation in hydrogen production. The archaeal functional proteins are mainly involved in methane metabolism in energy metabolism, such as acetyl-CoA decarboxylase, and methyl-coenzyme M reductase, and the acetic acid pathway exhibited the highest proportion of the total. The archaea of genus *Methanosarcina* in phylum *Euryarchaeota* can produce methane under the effect of multi-functional proteins through acetic acid, CO_2_ reduction, and methyl nutrient pathways. The study demonstrates metaproteomics as a new way of uncovering community functional and metabolic activity. The combined information was used to identify the metabolic pathways and organisms crucial for lignocellulosic biomass degradation and biogas production. This also regulates the process from its protein levels and improves the efficiency of biogas production using reed straw biomass.

## Introduction

To reduce the reliance on finite fossil fuels and mitigate concern over climate change, conversion of lignocellulosic biomass to energy sources such as biogas is currently receiving much research attention. China produces 7 billion tons of straw *per annum*, occupied for up to 30% of global straw yield, and more than 30% of this is wasted, so agriculture has beaten other industries to becoming the largest non-point source pollution industry in China. Due to traditional farming methods, people are unaware of the harm caused and frequently burnt the straw in the open-air, causing severe air pollution, and wasting resources [1]. In 2015, the National Development and Reform Commission published the *Notice on Further Accelerating the Comprehensive Utilisation of Straw and Prohibition of Straw Burning*. It points out that it is necessary to promote the orderly development of biomass gasification by using lignocellulosic biomass, including straw, as a raw material, to improve China’s rural energy structure.

Hydrogen as a clean energy source has characteristics such as: a high calorific value, it is clean, recyclable, efficient, and can be used in fuel cells. Owing to straw being rich in nutrients, cellulose, and hemicellulose, which contain 75% polysaccharide sugars, straw is an ideal hydrogen source [2]. During anaerobic fermentation, hydrolysing bacteria degrade macromolecular water-insoluble organic matter into soluble compounds, which are then degraded by hydrogenogens and acid-producing bacteria into micromolecular compounds consisting of hydrogen, volatile fatty acids (VFA), and alcohols [3]. Syntrophic bacteria and methanogens then metabolise to produce methane [4]. Combined hydrogen and methane production by anaerobic fermentation (CHMP-AF) provides a promising method for straw energy utilisation, which can effectively get rid of the feedback inhibition of hydrogen partial pressures and acidification. However, complex microbiologic populations vary dynamically in different stages of the CHMP-AF. Moreover, different environmental factors, operational, and control conditions significantly affect the microbial protein functions and metabolic pathways [5]. All these factors bring severe challenges to the regulation and stable operation of the CHMP-AF [6].

Traditional methods used in microbiology and next generation sequencing technologies were applied to deepen understanding of the constituents of microbial communities [7–9]; however, the functional genes fail to identify, and explain the correlation of functional proteins and metabolic activity in anaerobic fermentation for microorganisms. The metaproteomic analysis method combines metagenomics data, classification diversity, and functional diversity with the biological processes found in the natural environment [10]. This method provides a new way of identifying functional proteins in microorganisms and studying their metabolic activity in complex biological pathways [11]. Recently, it has been used in complex environments, such as lakes, oceans, and soils, which is a powerful tool for analysing phylogeny and the functions of microorganism populations [12–14]. However, metaproteome preliminary studies on the anaerobic fermentation process have been reported [15]. The dynamic analysis of microbial community structures and protein function expressions in the CHMP-AF and their interaction are rarely investigated.

This research studied the microbial community structure and functional succession during the CHMP-AF process based on a metaproteomic analysis for hydrogen and methane production under cellulase pretreatment of reed straw. The metaproteomic approach also gives functional insights into the CHMP-AF as a result of the identified protein sequences. The response correlation between key functional proteins and major metabolic activity of microorganisms was also investigated.

## Materials and methods

### Feedstock and inocula

Reed straw was collected from field monitoring station of Ulansuhai, which was in charge by Chinese Research Academy of Environmental Sciences, and gave the permission to conduct this study on the site. Reed straw was dried and ground, passed through a 20-mesh screen and stored at 4°C. The inoculation sludge was obtained from the reactor for swine manure anaerobic fermentation and filtered through a 30-mesh sieve to remove coarse particles. The characteristics of the substrate and inocula are summarised in Table 1.

**Table 1:** Characterisation of the raw materials and inocula.

Parameters	Reed straw	Inoculation sludge
**Total solids (TS, %)**	98.68	16.69
**Volatile solids (VS, %)**	91.39	11.97
**Carbon (%)**	33.77	35.20
**Nitrogen (%)**	0.57	3.06
**C /N ratio**	59.25	11.50
**Cellulose (%)**	28.04	—
**Hemi cellulose (%)**	16.77	—
**Lignin (%)**	14.65	—

### Experimental design

Batch experiments were performed in triplicate in 2 L bioreactors. Based on a previous study, the optimum hydrogen and methane production performance from reed straw were observed under the cellulase R-10 (Yakul, Japan) pretreatment and compared with acid and alkali pretreatments. Carrillo and Valldeperas reported that pretreatment of straw under alkaline condition will enhance its catalytic efficiency [16]. In this study, 32 g reed straw was mixed with 8% (w/v) cellulase R-10 aqueous solution and soaked for 48 h. The reactors were placed in an orbital shaker operating at 150 rpm at 48 ± 1°C since the optimal working condition for cellulase R-10 were: a pH of 4.5 to 6.5 and a temperature of 45–60°C. Some 200 mL inoculation sludge (with a VS _reed_ / VS _sludge_ ratio of 1.2:1) were added to each bioreactor. The total volume was increased to 1.6 L using distilled water. The initial pH was adjusted to 5.0 using 1 M HCl or NaOH before starting the experiment. The bioreactors were filled with nitrogen gas for 10 min to remove the oxygen and then placed in a water bath (containing a vibrator rotating at 150 rpm) at 37 ± 1°C. The control tests were prepared using reeds straw without pretreatment of cellulase at the same time.

The biogas samples were taken at six hourly intervals. The effluent samples were taken once a day for chemical index analyses. The mixed liquor for metaproteomics analyses was taken from bioreactors in four different stages, such as peak stage of hydrogen production (I, 6–12 h), late stage of hydrogen production (II, 69-93h), peak methanogenic stage (III, 89-113h), and late methanogenic stage (IV, 401-425h).

### Kinetic analysis

The cumulative volume of hydrogen and methane produced in the batch experiments can be calculated by the modified Gompertz equation:

$$H=P\cdot \exp\left\{-\exp\left[\frac{R_{\max_{}}\cdot e}{P}\left(\lambda -t\right)+1\right]\right\}$$ (1)

where H is the cumulative hydrogen or methane production (mL), *P* is the hydrogen or methane production potential (mL), *R*_*max*_ is the maximum hydrogen or methane production rate (mL/h), e is 2.72, *λ* is the lag-phase time (h) and t is the incubation time (h). The values of *P*, *R*_*max*_ and λ can be estimated using Origin 8.0 [17].

### Protein extraction and separation

Proteins were extracted from 50 mL samples using the method published by Benndorf *et al*. [18] and subsequently separated by one-dimensional sodium dodecyl sulphate-polyacrylamide gel electrophoresis (SDS–PAGE) as described previously [19]. The protein pellet was dissolved in 20 μL sample buffer for SDS-PAGE and incubated for 20 min at 100°C. After centrifugation, 5 μL of the supernatant was loaded onto SDS gel (4% stacking gel and 10% separating gel), which was stained with Coomassie brilliant blue R250, after electrophoresis. For identification of proteins from SDS-PAGE of different stages samples, the complete lanes were divided into bands and subjected to immediate in-gel tryptic digestion (Promega, Madison, WI, USA).

### LC-MS/MS analysis

Digested peptides were separated by nano-LC (Ultimate 3000, Dionex, Sunnyvale, CA, USA; trap column: Acclaim PePmap 100, C18, 3.0 μm, 75μm×2cm, 100A, Thermo Scientific, Pittsburgh PA, USA; column: Venusil×BPC, C18, 5.0 μm, 150A, Agela Technologies, Wilmington, DE, USA; eluent: 0.1% formic acid, 0% to 60% acetonitrile) and analysed by MS/MS (Q Exactive, Thermo Scientific, Pittsburgh PA, USA). Database searches were carried out using MS/MS ion search (MASCOT, http://www.matrixscience.com) against NCBInr. Protein matches were only accepted if they were identified by a minimum of one unique peptide.

### Metaproteomic analysis

All proteins were manually annotated with the aid of BLASTP (NCBI, http://blast.ncbi.nlm.nih.gov/Blast.cgi), and the protein hit that showed the highest sequence identity was recorded, including the identity of the protein and the name of the organism. Higher protein abundance is represented by a higher number of MS/MS spectra acquired from peptides of the respective protein. KEGG Orthology and Links Annotation (KOALA) is KEGG's internal annotation tool for K number assignment of KEGG GENES using SSEARCH computation. GhostKOALA assign K numbers to the user's sequence data by GHOSTX searches, against a nonredundant set of KEGG GENES. Thus, KOALA was used to annotate these metagenome sequences, which perform KO (KEGG Orthology) assignments from the proteins identification; it is and freely available at the KEGG Web site (http://www.kegg.jp/blastkoala/) [20].

### Analytical methods

Total solids, volatile solids and pH were measured according to the APHA standard methods [21]. The percentage compositions of lignin, cellulose and hemicellulose were determined according to the VanSoest method [22]. The cumulative biogas production was examined by a Milli Gascounter (Ritter MGC-1, Germany).

The composition of the biogas (i.e., H_2_, CH_4_ and CO_2_) was measured by a gas chromatograph (Perkin Elmer Clarus 500, USA) equipped with a thermal conductivity detector and a 2-m high-porosity polymer bead-packed column. The operating temperatures of the injection port, oven and detector were set to 50, 150 and 150°C, respectively. Argon was used as the carrier gas at a flow rate of 40 mL/min. The VFA and ethanol concentration were determined using a gas chromatograph (Shimadzu GC-2010, Japan) equipped with a flame ionisation detector and a 30 m×0.25 mm×0.25 mm fused-silica capillary column (Agilent DB-VRX, USA). The injection temperature was 200°C. The oven temperature was initially set to 40°C and increased to 220°C thereafter at a rate of 9°C per minute. Helium was used as the carrier gas at a flow rate of 1.2 mL/min and a split to a column flow ratio of 10:1.

## Results

### Biogasification performance and kinetic analysis

**Fig 1:**
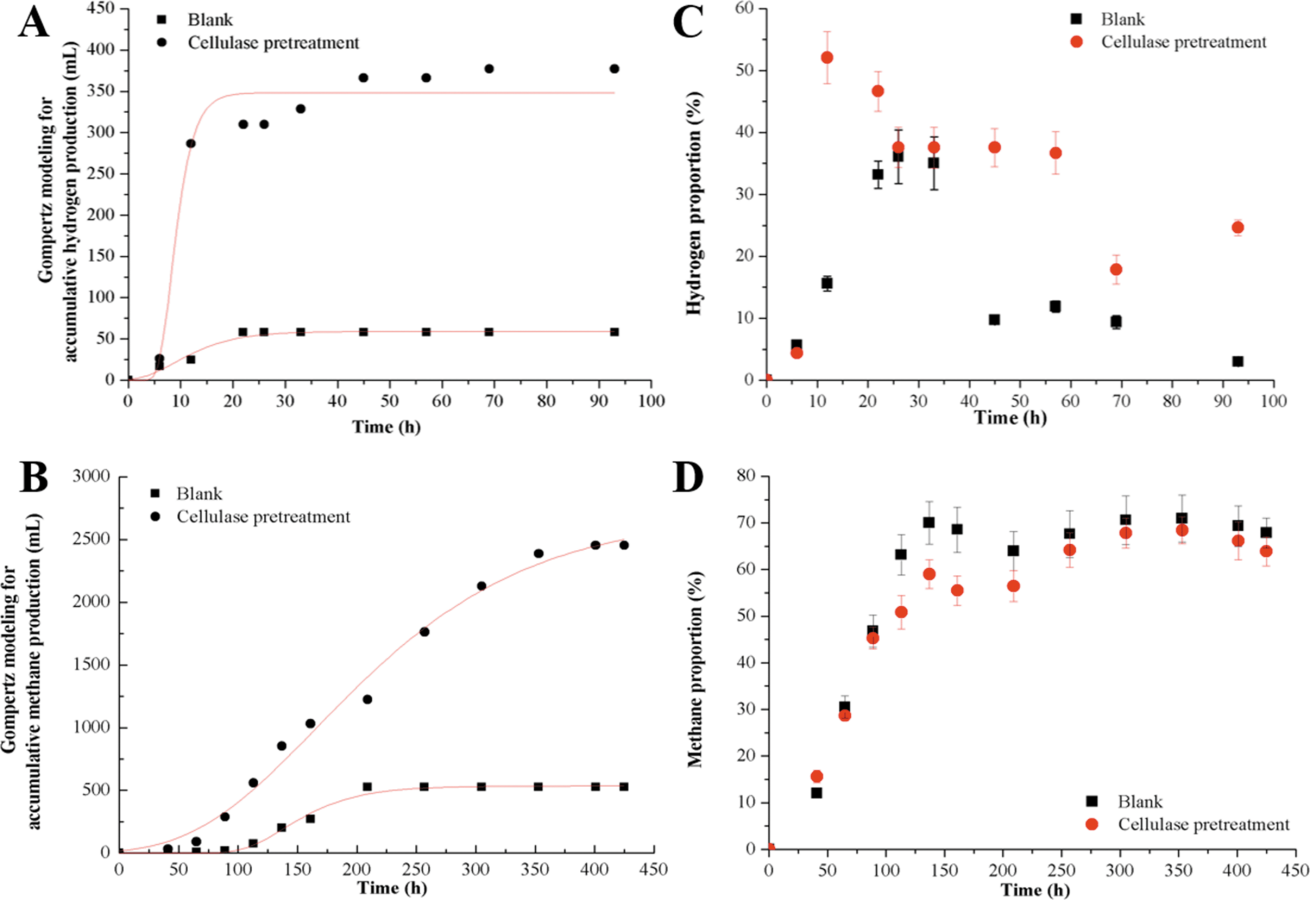
The performance of hydrogen and methane cumulative production and proportion based on the kinetic analysis in the CHMP-AF. The hydrogen cumulative production (A) and methane cumulative production (B) with the time course were analyzed by Gompertz modeling. The hydrogen proportion (C) and methane proportion (D) identified by gas chromatograph during the CHMP-AF process.

The kinetic analysis and biogas proportion are shown in Fig 1 and Table 2. The performance of hydrogen and methane production from reed straw pre-treated with cellulase was significantly improved in the CHMP-AF. For the cellulase pretreatment groups, the cumulative hydrogen production increased rapidly in the first 12 h, and then increased slowly thereafter in the hydrogen production stage. The maximum hydrogen production potential and hydrogen production rate reached 348.32 mL and 54.03 mL/h, respectively, which were 5.9 and 15.8 times that of the control. The highest hydrogen proportion was 52.1% at 12 h. In the methanogenic stage, the cumulative methane production in the cellulase pretreatment groups increased with time. The maximum methane production potentials and methane production rate reached 2709.94 mL and 9.71 mL/h, respectively, and were 5.1 and 1.7 times that of the control. The maximum methane proportion was 68.4%. Moreover, the lag-phase time (63.1 h) was approximately two times shorter under cellulose pretreatment than that of the control (104.5 h) in the methanogenic stage, which is the rate-limiting step in the CHMP-AF. This result indicates that the cellulose pretreatment result in a quick start-up in the methanogenic stage and enhanced the biogasification performance in the CHMP-AF.

**Table 2:** Kinetic coefficients for the CHMP-AF in the hydrogen production and methanogenic stages.

	Hydrogen production stage				Methanogenic stage			
	*P* (mL)	*R*_*max*_ (mL/h)	*λ* (h)	R^2^	*P* (mL)	*R*_*max*_ (mL/h)	*λ* (h)	R^2^
**Blank**	59.18	3.43	2.6	0.964	535.21	5.76	104.5	0.990
**Cellulase pretreatment**	348.32	54.03	5.8	0.961	2709.94	9.71	63.1	0.991

*P* is the hydrogen/methane production potential, *R*_*m*_ is the maximum hydrogen/methane production rate, λ is the lag-phase time and R^2^ is the determination coefficient.

### Phylogenetic analysis

By using the metaproteomic method, microbial proteins in reed straw pre-treated with cellulose in different stages (I, II, III, and IV stages) of the CHMP-AF were identified, thus obtaining 935 bacterial proteins and 375 archaeal proteins. The proteins in the methanogenic stage were more numerous than those in the hydrogen production stage.

#### Bacterial community structure analysis

**Fig 2:**
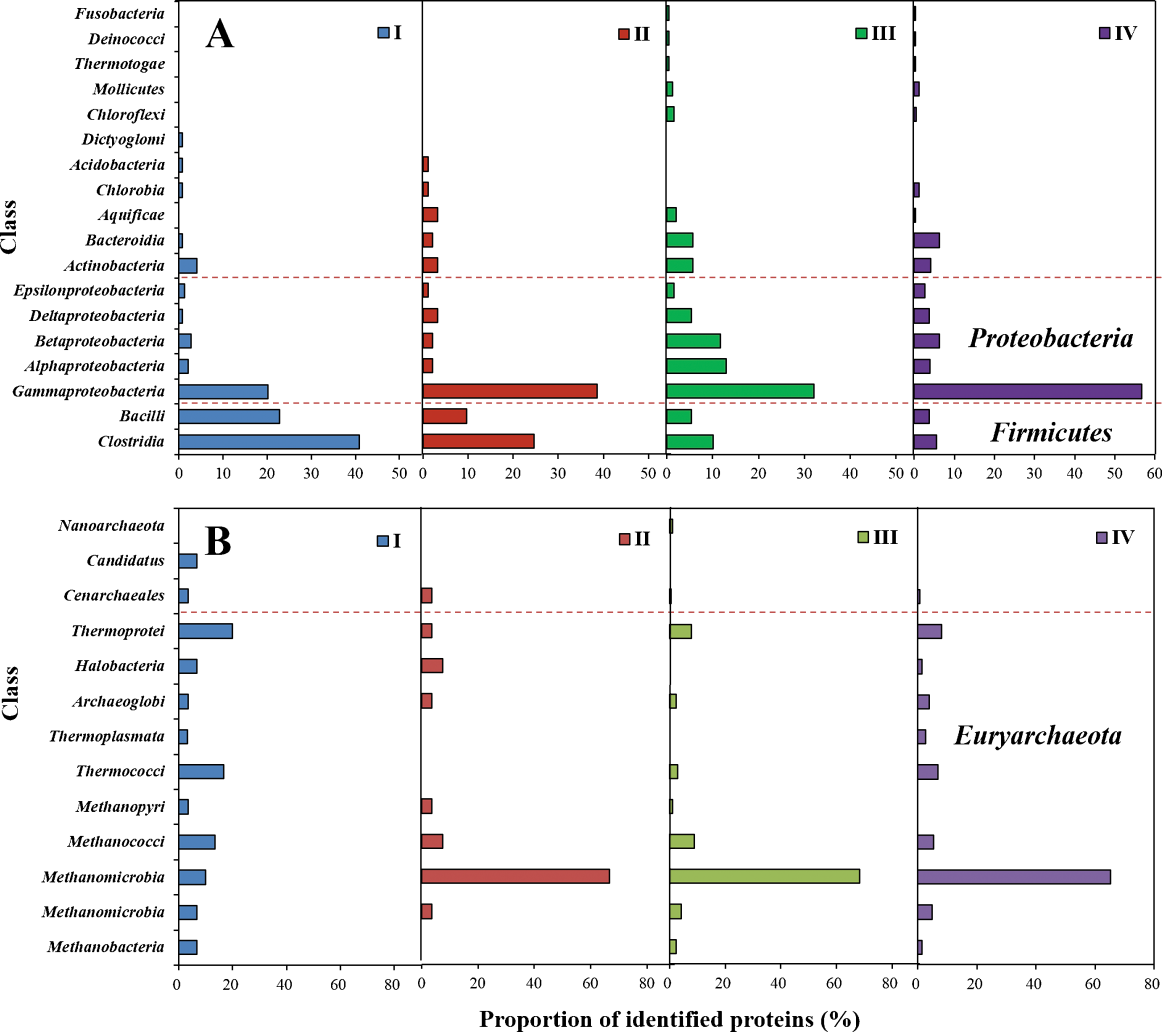
Assessment of microbial community composition in different stages at the classes taxonomic level. (A) Bar graph showing the proportion of bacterial identified proteins in class’s level and divided into three parts according to the phyla. (B) Bar graph showing the proportion of archaea identified proteins in class’s level and divided into three parts according to the phyla.

The compositions of bacterial populations at different stages of the CHMP-AF are shown in Fig 2A and S1 Table. The results demonstrate that the bacterial populations are mainly in phyla *Firmicutes*, *Proteobacteria*, *Actinobacteria* and *Bacteroidetes*. In the peak stage of hydrogen production, classes *Clostridia* and *Bacilli* in phylum *Firmicutes* accounted for 40.9% and 22.8% of the total, while they gradually decreased thereafter. Compared with the hydrogen production stage, the proportion of phyla *Proteobacteria* and *Bacteroidetes* increased in the methanogenic stage and classes *Gammaproteobacteria* and *Epsilonproteobacteria* reached 56.8% and 2.7%, respectively, in the late stage. Furthermore, classes *Alphaproteobacteria*, *Betaproteobacteria* and *Deltaproteobacteria* show the highest proportions in the peak methanogenic stage, accounting for 13%, 11.7%, and 5.3%, respectively, while class *Bacteroidetes* reaches 6.2% in the late methanogenic stage. The proportion of class *Actinobacteria* stabilised in the range between 3.2% to 5.7%.

Genus *Clostridium* in class *Clostridia* accounted for the largest proportion in the hydrogen production stage. Genus *Caldicellulosiruptor* in class *Clostridia* was omnipresent and could degrade cellulose and showed good thermal stability, which was important for the synergistic hydrolysis of insoluble cellulose [23]. In the hydrogen production stage, genus *Acidobacteria*, belonging to the acidophilus type, was detected. This can adapt to the acidic environment during hydrogen production and plays an important role in maintaining a stable anaerobic system. In the late stage of hydrogen production, the proportion of class *Gammaproteobacteria* increased to 38.7% in which genus *Klebsiella* is a microorganism commonly seen in anaerobic fermentation systems. It is reported that bacterium *Klebsiella* sp. HE1 is able to produce hydrogen and can also synthesise methane by using hydrogen under specific conditions [24]. In the peak methanogenic stage, genus *Escherichia*, as the facultative anaerobes, accounting for 25% overall, fermented the glucose and other sugars present to produce pyruvic acid, which was then further transferred to VFA and hydrogen [25,26]. As the methanogenic process ended, the proportion of genus *Escherichia* decreased to 8.9% in the late methanogenic stage.

#### Archaea community structure analysis

Fig 2B and S2 Table shown the compositions of archaeal populations in different stages of the CHMP-AF. The results show that phyla *Euryarchaeota*, *Crenarchaeota* and *Thaumarchaeota* were dominant in archaeal populations and a few representatives from phyla *Korarchaeota* and *Nanoarchaeota* were found in the stages of hydrogen and methane production, respectively. Class *Thermoprotei* in phylum *Crenarchaeota* in the peak stage of hydrogen production accounted for the highest proportion (20%), followed by classes *Thermococci* (16.7%) and *Methanococci* (13.3%). Class *Methanomicrobia* accounted for the highest proportion in the methanogenic stage and the proportions in the peak, and late, methanogenic stages were 68.3% and 65.1%, respectively. During methane production, the proportion of classes *Thermococci*, and *Thermoprotei* gradually decreased and class *Nanoarchaeota* only appeared in the peak methanogenic stage, and accounted for 1.2% overall.

The study shows that genus *Methanosphaera* in class *Methanobacteria* belongs to the archaea and produces methane as a specific metabolite under anaerobic conditions. It can generate methane with hydrogen and methanol. Genus *Pyrococcus*, containing a soluble HD containing nickel, can generate hydrogen and CO_2_ by using carbohydrates and proteins. Genus *Methanosarcina* in class *Methanomicrobia* not only produces methane by utilising acetic acid in the late stage of hydrogen production, but is also autotrophic as a result of its use of methanol and hydrogen [27]. In addition, genus *Methanosarcina* presents the largest number and proportion of its functional proteins. Therefore, it is the dominant microorganism in the methanogenic stage during the CHMP-AF process.

### Metaproteomics analysis

#### Bacterial protein function analysis of different stages

**Fig 3:**
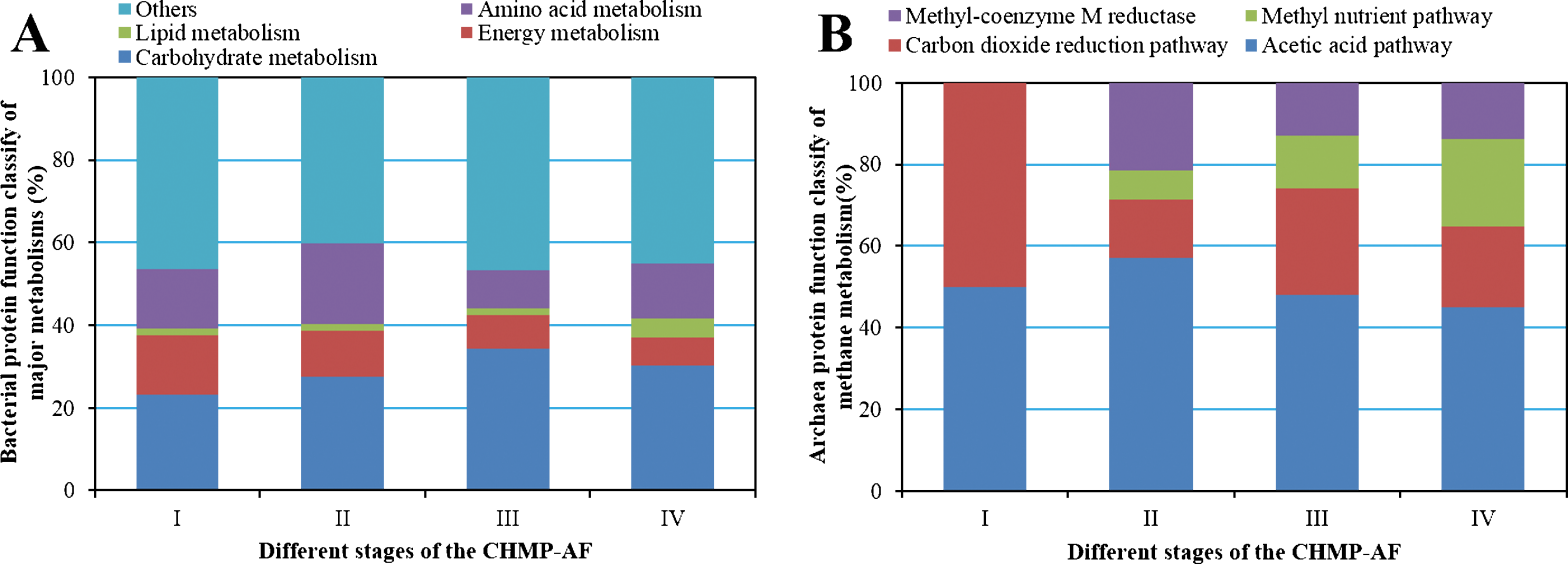
Protein function classify of bacterial major metabolisms and archaea methane metabolism in different stages of the CHMP-AF. (A) Bar graph showing the proportion of identified bacterial proteins involved into the carbohydrate, energy, lipid, amino acid, and other metabolisms. (B) Bar graph showing the proportion of identified archaea proteins involved into the methane metabolism by acetic acid pathway, carbon dioxide pathway, methyl nutrient pathway and methyl-coenzyme M reductase, respectively.

The metaproteomics analysis demonstrates that the bacterial proteins present in the process of CHMP-AF have diverse functions [28]. The functional proteins involved in the carbohydrate metabolism accounted for the largest proportion of the total, and were higher in the methanogenic stage than in the hydrogen production stage, followed by proteins related to amino acid and energy metabolism, as shown in Fig 3A.

The hydrogen production pathway was mainly involved in the metabolism of pyruvic acid and glycolysis in carbohydrate metabolism. In the peak stage of hydrogen production, it was found that L-lactate dehydrogenase, involved in pyruvic acid metabolism to produce lactic acid, came from bacteria in genus *Streptococcus*. Pyruvate-flavodoxin oxidoreductase and uncharacterized protein YdiJ which can synthesise acetyl-CoA through the electron transfer from pyruvic acid and is the most important enzyme for hydrogen production from pyruvic acid, came from genera *Klebsiella* and *Escherichia* in class *Gammaproteobacteria*. NADH-quinone oxidoreductase subunit B, which binds the 4Fe-4S cluster, and is involved in proton and energy transfers in the respiratory chain, was a hub for electron transfer between respiratory chain complexes and came from genus *Xylella* in class *Gammaproteobacteria*. In the late stage of hydrogen production, isocitrate dehydrogenase and aconitate hydratase, involved in the tricarboxylic acid cycle, and malate synthase G1 and NAD-dependent malic enzyme, taking part in the aldehyde acid cycle, were added to the carbohydrate metabolism process and came from bacteria in genera *Azotobacter*, *Pseudomonas and Sodalis* in class *Gammaproteobacteria*.

The amounts of functional proteins in bacteria significantly increased in the methanogenic stage and the proportion of functional proteins involved in carbohydrate metabolism also increased. Moreover, those functional proteins relating to glycolytic pathways increased. In the peak methanogenic stage, it was found that pyruvate dehydrogenase E1 component, involved in pyruvate metabolism, can transform pyruvic acid into acetyl-CoA and CO_2_ and came from genus *Mycobacterium* in class *Actinobacteria* and genera *Enterobacter* and *Pseudomonas* in class *Proteobacteria*. In this stage, acetate kinase from genera *Halothermothrix*, *Syntrophomonas and Clostridium* in class *Clostridia* was the important enzyme necessary for acetyl-CoA to generate acetic acid and played a linking role in the process of combined hydrogen and methane production [29]. Furthermore, there remained a certain amount of pyruvate-flavodoxin oxidoreductase and NADH-quinone oxidoreductase subunit B present. In the late methanogenic stage, the numbers of methyltransferase enzymes, pyruvate-flavodoxin oxidoreductase and NADH-quinone oxidoreductase increased. Ferredoxin-NADP reductase was an important enzyme involved in the balanced regulation of NADH/NAD^+^ and came from genus *Azotobacter* in class *Proteobacteria*. Methylenetetrahydrofolate-tRNA-(uracil-5-)- methyltransferase TrmFO, as an important enzyme taking part in methane production with CO_2_, can catalyse the tetrahydrofolate and came from genus *Bacillus* in class *Firmicutes*. Moreover, in the methanogenic stage, those proteins relating to ABC transfer increased significantly and the energy of ATP hydrolysis can be adopted to realise transmembrane transport of sugars, amino acids, metal ions, peptides, proteins, and cellular metabolites [12]. Compared with the hydrogen production stage, more diversified and active microbial populations and proteins were involved in material transport and metabolism. While, the proteins involved in carbohydrate metabolism, energy metabolism, lipid metabolism, and amino acid metabolism took up relatively stable proportions of the total.

#### Archaea protein function analysis in different stages

According to the functional analysis, the detected archaeal proteins were mainly involved in methane metabolism in energy metabolism and accounted for 6.67% (I), 45.16% (II), 31.58% (III), and 34.23% (IV), respectively, during the CHMP-AF process. The functional proteins involved in amino acid metabolism, carbohydrate metabolism, biosynthesis, and transport were also included. This research mainly analysed the functional proteins of archaea involved in methane metabolism at different stages (Fig 3B).

In the peak stage of hydrogen production, only a few of functional proteins were involved in methane metabolism. Acetyl-CoA decarboxylase is an important enzyme for methane production using acetic acid and it came from genus *Methanosarcina* in class *Methanomicrobia*. 5,10-methylenetetrahydromethanopterin reductase catalyses diiodomethane-tetrahydromethanopterin into methyl-H_4_MPT, while H_4_MPT, derived from tetrahydrofolate, is used to transfer carbon across the levels of methyl, methylene, and methyl [30]. They are important enzymes for the methane production pathway using CO_2_ and from archaea in genus *Methanobacteriales* in class *Methanobacteria*. In the late stage of hydrogen production, the number of functional proteins involved in the metabolism of methane production increased. Acetyl-CoA decarbonylase accounted for the largest proportion in the methane production pathway based on acetic acid, followed by tetrahydromethanopterin S-methyltransferase subunit H and F_420_-methylenetetrahydromethanopterin dehydrogenase from genera *Methanosarcina* and *Methanoculleus* in class *Methanomicrobia* as involved in methane production by reducing CO_2_ [30]. The results show that coenzyme F_420_, as a deazaflavin analogue, is used as an electron acceptor of hydrogenase, formate dehydrogenase and carbon monoxide dehydrogenase, as well as an electron donor for reductase NADP^+^; moreover, it utilises H_2_ and formic acid as electron donors to produce methane by reducing CO_2_ [31,32]. The methanol-corrinoid protein co-methyltransferase came from genus *Methanosarcina* through involvement in the methyl nutrient type of methane production pathway, methanol-corrinoid protein co-methyltransferase can produce methane by reducing methyl with H_2_ in methyl compounds, or produce methane through the dismutation of methyl compounds from genus *Methanosarcina*. In addition, methyl-coenzyme M reductase, catalysed by the reductase of methyl coenzyme M containing nickel, reduces CH_3_-S-CoM to produce methane and Co B-S-S-CoM by using HS-CoB as the direct electron donor [33]. This enzyme, present in genus *Methanos arcina*, is the terminal methyl carrier and the critical enzyme for methane production.

In the methanogenic stage, the number of functional proteins of archaea involved in the metabolism of methane production increased and the proportion stabilised at between 31.58% and 34.23%. In the peak methanogenic stage, acetyl-CoA decarbonylase still accounted for the largest proportion of enzymes involved in the acetic acid type of methane production pathway. The species of archaea participating in methane production by utilising the CO_2_ reduction pathway increased and the F_420_-dependent methylenetetrahydromethanopterin dehydrogenase came from genera *Methanosarcina*, *Methanoculleus* and *Methanosphaerula*. Moreover, coenzyme F_420_ hydrogenase (sub-unit beta) and coenzyme F_420_ hydrogenase subunit alpha came from genera *Methanococcales* and *Methanosarcina*, respectively. The 5,10-methylenetetrahydromethanopterin reductase came from genera *Methanobacteriales* and *Methanosarcina*. Formate dehydrogenase subunit alpha is an important enzymefor formic aciddehydrogenation came from genus *Methanococcales* in class *Methanococci*. The number of functional proteins involved in methane production through the methyl nutrition pathway increased. Monomethylamine corrinoid protein 1, monomethylamine methyltransferase MtmB, trimethylamine corrinoid protein 1, and methanol-corrinoid protein co-methyltransferase which use methylamine, trimethylamine, and methanol as the substrates for methane production, and their functional proteins all came from genus *Methanosarcina*. In the late stage of methane production, similar functional proteins of archaea were involved in the metabolism for methane production, among which functional proteins relating to methyl nutrient types of methane production showed constantly increasing proportions. In this stage, dimethylamine corrinoid protein 2 also appeared; this can use dimethylamine as a methyl donor to produce methane and is from those archaea in genus *Methanosarcina*.

## Discussions

**Fig 4:**
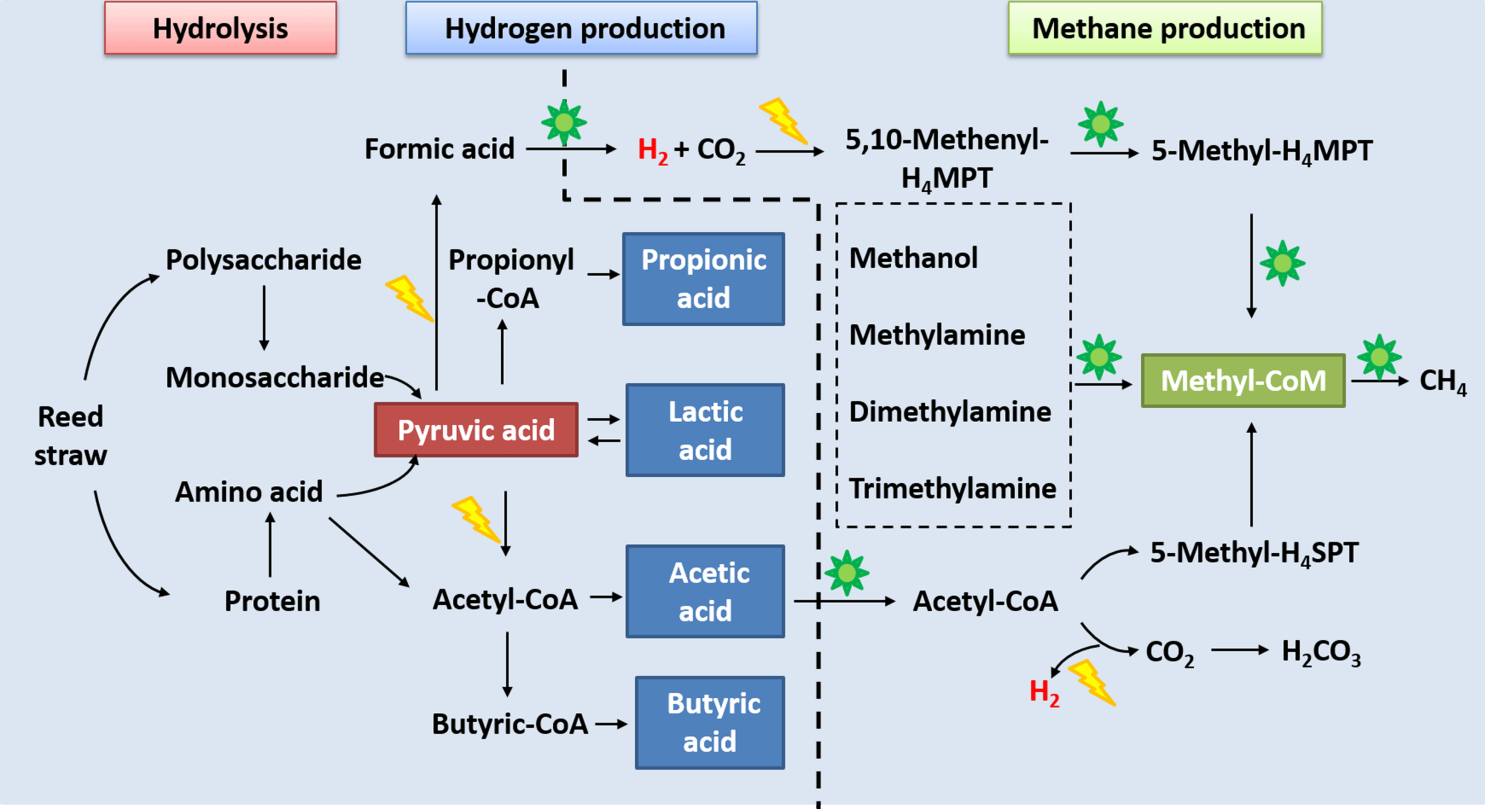
Depiction of the metabolic characteristics of functional proteins and metabolites in different stages inferred from the metaproteome. The yellow lightning is the identified protein from bacterial community. The green sun is the identified protein from archaea community.

The CHMP-AF is a biochemical process in which substrates, enzymes, and microorganisms interact with, and inhibit, each other (Fig 4). Through hydrolysis, macromolecular polysaccharides in reed straws were hydrolysed into a monosaccharide, and proteins into amino acids, which were then transformed to pyruvic acid through the glycolytic pathway. Pyruvic acid can realise transfers among sugar, fats, and amino acids through the acetyl-CoA, and tricarboxylic acid cycles and it is an important hub in the metabolism of the three nutrient substances. The classic theory of hydrogen production using microorganisms is the hydrogen production process that uses pyruvic acid as direct, or indirect, electron donors. This theory includes hydrogen production through decarboxylation of pyruvic acid and formic acid decomposition as well as that hydrogen production theory of NADH/NAD^+^ equilibrium regulation proposed by Tanisho, *et al*. [34]. The results of this research showed that the pyruvate dehydrogenase E1 component, in functional proteins relating to hydrogen production, is involved in the pyruvic acid decarboxylase pathway for hydrogen production [35]. Formate dehydrogenase subunit alpha came from genus *Methanococcales*., which can catalyse the dehydrogenation of formic acid and decompose formic acid to produce hydrogen and CO_2_ under the common effects of hydrogenase [36]. Ferredoxin-NADP reductase, present in bacteria in genus *Azotobacter*, is an important enzyme for NADH/NAD^+^ equilibrium regulation in hydrogen production. NADH can couple with the fermentation processes of propionic acid, butyric acid, ethanol, or lactic acid and therefore may be oxidised as NAD^+^ to produce hydrogen, thus ensuring an NADH/NAD^+^ equilibrium in the metabolism [25]. Therefore, many VFAs were found in the later stages of hydrogen production and they were further decomposed into CO_2_ and one-carbon compounds by microorganisms to be reduced for methane production [4].

Methane production from microorganisms is a process able to reduce methyl in CO_2_ and one-carbon compounds to methane under the common effects of many enzymes and coenzymes in one-carbon metabolism. Coenzymes, closely relating to methane production, can be divided into two categories: one-carbon carriers, including H_4_MPT, H_4_SPT and coenzyme M; the other is electron carriers comprising ferredoxin, coenzyme F_420_, coenzyme B and cytochrome [37]. The results demonstrate thatthree different pathways for methane production are available in the CHMP-AF using reed straw. This was cooperatively realised by a large number of archaea proteins and a small number of bacterial proteins, with microorganic differences and diversity in different stages.

The functional proteins relating to methane production through the acetic acid pathway exhibited the highest proportion of the total. Acetyl-CoA decarboxylase uses acetate as its only carbon source and for the energy required to catalyse the decarboxylation of acetyl CoA to decompose to CO_2_. Carboxyl is oxidised to produce electron donating H_2_ for the methyl reduction and methane generation [35]. This pathway is mainly achieved by genus *Methanosarcina* and a few archaea of genus *Methanosaeta*. The methyl nutrient pathway is active in the methanogenic stage. This study detected many functional proteins by using methanol, methylamine, dimethylamine, and trimethylamine as its substrates. Under the actions of methyl-coenzyme M reductase of the methyl carrier, methane was produced at the end of the methane production stage through that pathway using the participation of genus *Methanosarcina*. Formic acid was decomposed by formate dehydrogenase subunit alpha to hydrogen and CO_2_, the latter of which was reduced under catalysis with ferredoxin-NADP reductase in bacterial protein to generate 5,10-methenyl-H_4_MPT, and then 5-methyl-H_4_MPT was generated due to the catalysis of 5, 10-methylenetetrahydromethanopterin reductas*e* of those archaeal proteins present [30]. Finally, methane was generated under the effects of methyl-coenzyme M reductase [33,38]. It can be seen that many bacteria and archaeal proteins were involved in methane production by using the CO_2_ reduction pathway, mainly including bacteria of genus *Azotobacter* and archaea of genera *Methanosarcina*, *Methanoculleus*, *Methanothermobacter* and *Methanocaldococcus*.

## Conclusion

The biogasification performance significantly improved and the functions and metabolic activity of microbial communities played significant roles during the CHMP-AF after cellulase pretreatment of reed straw. The metaproteomic analysis revealed that bacterial functional proteins, such as ferredoxin-NADP reductase, acetate kinase, and NADH-quinone oxidoreductase, mainly belonging to phyla *Firmicutes*, *Proteobacteria*, *Actinobacteria* and *Bacteroidetes*, are involved in carbohydrate metabolism, energy metabolism, lipid metabolism, and amino acid metabolism. The archaeal functional proteins are mainly involved in methane metabolism in energy metabolism, such as acetyl-CoA decarboxylase, and methyl-coenzyme M reductase, and the acetic acid pathway exhibited the highest proportion of the total. The genus *Methanosarcina* in phylum *Euryarchaeota* present the highest functional diversity in methane metabolism and can produce methane under the influence of multi-functional proteins through acetic acid, CO_2_ reduction, and methyl nutrient pathways. Therefore, the functional diversity and metabolic activity of microbial communities can be combined with the metabolic pathway during the CHMP-AF to regulate from its protein levels and improve the hydrogen and methane production potential using a lignocellulosic biomass such as reed straw.

## Supporting information

S1 Table Bacterial community structure based on the metaproteomics analysis. I is the peak stage of hydrogen production. II is the late stage of hydrogen production. III is the peak methanogenic stage. IV is the late methanogenic stage. % is the proportion of the identified bacterial proteins in different stages of the CHMP-AF.
(DOCX)

S2 Table Archaea community structure based on the metaproteomics analysis. I is the peak stage of hydrogen production. II is the late stage of hydrogen production. III is the peak methanogenic stage. IV is the late methanogenic stage. % is the proportion of the identified archaea proteins in different stages of the CHMP-AF.
(DOCX)

S1 Data The bacteria protein sequencing data files based on the metaproteomics analysis. (RAR)

S2 Data The archaea protein sequencing data files based on the metaproteomics analysis. (RAR)
