# Supplementary material for: Metaproteomics analysis of the functional insights into microbial communities of combined hydrogen and methane production by anaerobic fermentation from reed straw
Source: PLoS One. 2017 Aug 17;12(8):e0183158. doi: 10.1371/journal.pone.0183158 (PMC5560556; doi:10.1371/journal.pone.0183158)
Supplement: S1 Table — I is the peak stage of hydrogen production. II is the late stage of hydrogen production. III is the peak methanogenic stage. IV is the late methanogenic stage. % is the proportion of the identified bacterial proteins in different stages of the CHMP-AF. (DOCX) [file pone.0183158.s001.docx]

**S1 Table. Bacterial community structure based on the metaproteomics analysis.** I is the peak stage of hydrogen production. II is the late stage of hydrogen production. III is the peak methanogenic stage. IV is the late methanogenic stage. % is the proportion of the identified bacterial proteins in different stages of the CHMP-AF.

| **Bacterial phyla** | **Classes** | **I** | | **II** | | **III** | | **IV** | |
| --- | --- | --- | --- | --- | --- | --- | --- | --- | --- |
|  |  | **Number** | **%** | **Number** | **%** | **Number** | **%** | **Number** | **%** |
| *Firmicutes* | *Clostridia* | 61 | 40.9 | 23 | 24.7 | 25 | 10.1 | 24 | 5.5 |
|  | *Bacilli* | 34 | 22.8 | 9 | 9.7 | 13 | 5.3 | 16 | 3.7 |
| *Proteobacteria* | *Alphaproteobacteria* | 3 | 2 | 2 | 2.2 | 32 | 13 | 17 | 3.9 |
|  | *Betaproteobacteria* | 4 | 2.7 | 2 | 2.2 | 29 | 11.7 | 27 | 6.2 |
|  | *Gammaproteobacteria* | 30 | 20.1 | 36 | 38.7 | 79 | 32 | 248 | 56.8 |
|  | *Deltaproteobacteria* | 1 | 0.7 | 3 | 3.2 | 13 | 5.3 | 13 | 3.7 |
|  | *Epsilonproteobacteria* | 2 | 1.3 | 1 | 1.1 | 4 | 1.6 | 14 | 2.7 |
| *Actinobacteria* | *Actinobacteria* | 6 | 4 | 3 | 3.2 | 14 | 5.7 | 18 | 4.1 |
| *Bacteroidetes* | *Bacteroidia* | 1 | 0.7 | 2 | 2.2 | 14 | 5.7 | 27 | 6.2 |
| *Aquificae* | *Aquificae* | -- | -- | 3 | 3.2 | 5 | 2 | 1 | 0.2 |
| *Chlorobi* | *Chlorobia* | 1 | 0.7 | 1 | 1.1 | -- | - | 5 | 1.1 |
| *Acidobacteria* | *Acidobacteria* | 1 | 0.7 | 1 | 1.1 | -- | -- | -- | -- |
| *Dictyoglomi* | *Dictyoglomi* | 1 | 0.7 | -- | -- | -- | -- | -- | -- |
| *Chloroflexi* | *Chloroflexi* | -- | -- | -- | -- | 4 | 1.6 | 2 | 0.5 |
| *Tenericutes* | *Mollicutes* | -- | -- | -- | -- | 3 | 1.2 | 5 | 1.1 |
| *Thermotogae* | *Thermotogae* | -- | -- | -- | -- | 1 | 0.4 | 1 | 0.2 |
| *Deinococci* | *Deinococci* | -- | -- | -- | -- | 1 | 0.4 | 1 | 0.2 |
| *Fusobacteria* | *Fusobacteria* | -- | -- | -- | -- | 1 | 0.4 | 1 | 0.2 |
