# Supplementary material for: Metaproteomics analysis of the functional insights into microbial communities of combined hydrogen and methane production by anaerobic fermentation from reed straw
Source: PLoS One. 2017 Aug 17;12(8):e0183158. doi: 10.1371/journal.pone.0183158 (PMC5560556; doi:10.1371/journal.pone.0183158)
Supplement: S2 Table — I is the peak stage of hydrogen production. II is the late stage of hydrogen production. III is the peak methanogenic stage. IV is the late methanogenic stage. % is the proportion of the identified archaea proteins in different stages of the CHMP-AF. (DOCX) [file pone.0183158.s002.docx]

**S2 Table. Archaea community structure based on the metaproteomics analysis.** I is the peak stage of hydrogen production. II is the late stage of hydrogen production. III is the peak methanogenic stage. IV is the late methanogenic stage. % is the proportion of the identified archaea proteins in different stages of the CHMP-AF.

| **Archaea phyla** | **Classes** | **I** | | **II** | | **III** | | **IV** | |
| --- | --- | --- | --- | --- | --- | --- | --- | --- | --- |
|  |  | **Number** | **%** | **Number** | **%** | **Number** | **%** | **Number** | **%** |
| *Euryarchaeota* | *Methanobacteria* | 2 | 6.7 | 0 | 0 | 4 | 2.4 | 2 | 1.3 |
|  | *Methanomicrobia* | 2 | 6.7 | 1 | 3.7 | 7 | 4.2 | 7 | 4.7 |
|  | *Methanomicrobia* | 3 | 10.0 | 18 | 66.7 | 114 | 68.3 | 97 | 65.1 |
|  | *Methanococci* | 4 | 13.3 | 2 | 7.4 | 15 | 9.0 | 8 | 5.4 |
|  | *Methanopyri* | 1 | 3.3 | 1 | 3.7 | 2 | 1.2 | 0 | 0 |
|  | *Thermococci* | 5 | 16.7 | 0 | 0 | 5 | 3.0 | 10 | 6.7 |
|  | *Thermoplasmata* | 1 | 3.3 | 0 | 0 | 0 | 0 | 4 | 2.6 |
|  | *Archaeoglobi* | 1 | 3.3 | 1 | 3.7 | 4 | 2.4 | 6 | 3.9 |
|  | *Halobacteria* | 2 | 6.7 | 2 | 7.4 | 0 | 0 | 2 | 1.3 |
| *Crenarchaeota* | *Thermoprotei* | 6 | 20.0 | 1 | 3.7 | 13 | 7.8 | 12 | 8 |
| *Thaumarchaeota* | *Cenarchaeales* | 1 | 3.3 | 1 | 3.7 | 1 | 0.6 | 1 | 0.7 |
| *Korarchaeota* | *Candidatus* | 2 | 6.7 | 0 | 0 | 0 | 0 | 0 | 0 |
| *Nanoarchaeota* | *Nanoarchaeota* | 0 | 0 | 0 | 0 | 2 | 1.2 | 0 | 0 |
